# Transnasal–Transoral Endoscopic Surgery Followed by Radiotherapy for Nasopharyngeal Spindle Cell Carcinoma

**DOI:** 10.7759/cureus.103001

**Published:** 2026-02-04

**Authors:** Yuji Kanazawa, Yoshiharu Kitani, Aya Matsubara, Riki Goda, Makoto Suzuki

**Affiliations:** 1 Department of Otolaryngology-Head and Neck Surgery, Shizuoka General Hospital, Shizuoka, JPN; 2 Department of Pathology, Shizuoka General Hospital, Shizuoka, JPN

**Keywords:** chronic kidney disease, endoscopic surgery, nasopharyngeal cancer, spindle cell carcinoma, transnasal, transoral

## Abstract

Spindle cell carcinoma (SpCC) is a rare and aggressive variant of squamous cell carcinoma characterized by the coexistence of epithelial and mesenchymal components, accounting for <1% of all head and neck malignancies. Nasopharyngeal SpCC is extremely rare, and its optimal management remains unclear, particularly in patients who cannot tolerate extensive surgery. An 80-year-old man presenting with right-sided ear fullness was diagnosed with nasopharyngeal SpCC (T1N0M0). Imaging revealed a submucosal tumor on the posterior wall of the nasopharynx that was in contact with the right Eustachian tube. The patient also had severe chronic kidney disease; therefore, conventional open approaches requiring prolonged operative time and significant surgical stress were considered unsuitable. Therefore, a combined transnasal-transoral endoscopic approach was selected to achieve maximal tumor reduction while minimizing surgical invasiveness. This approach provides a wide and complementary surgical view of the nasopharynx, enabling precise dissection of tumor margins extending cranially and caudally beyond the soft palate. Intraoperatively, the Eustachian tube was clearly visualized and confirmed to be intact. Histopathological examination revealed SpCC with a positive surgical margin on the posterior nasopharyngeal wall. Postoperative radiotherapy was administered to the surgical bed with positive margins. The patient achieved disease-free survival for >3 years after surgery, with stable perioperative renal function. This case highlights the advantages of the transnasal-transoral endoscopic approach in providing adequate visualization and access to the nasopharynx while reducing surgical morbidity. The combination of maximal cytoreductive endoscopic surgery and postoperative radiotherapy may represent an effective and less invasive treatment option for nasopharyngeal SpCC, particularly in patients with significant comorbidities.

## Introduction

Spindle cell carcinoma (SpCC) is a rare neoplasm characterized by the concurrent presence of mesenchymally differentiated spindle-shaped cells and squamous cell carcinoma (SCC) components. This carcinoma accounts for <1% of all head and neck cancers, with the larynx being the most frequently affected site [1,2]. Compared with SCC, SpCC is considered a more aggressive malignancy and poses unique therapeutic challenges, including lower radiosensitivity and a higher rate of local recurrence [2]. Consequently, a higher proportion of patients with SpCC are treated surgically rather than with radiotherapy, in contrast to patients with head and neck SCC. However, due to the rarity of nasopharyngeal SpCC and the limited number of reported cases, standardized treatment protocols have not been established, and optimal management remains unclear, particularly in patients who cannot tolerate extensive surgery because of comorbidities.

Herein, we report the case of an 80-year-old man with nasopharyngeal SpCC. Surgical access to the nasopharynx is technically challenging and highly invasive when using conventional open approaches such as the maxillary swing, midfacial degloving, and transmaxillary or infratemporal fossa approaches. Owing to the patient’s poor tolerance for prolonged surgery due to severe renal impairment, palliative tumor resection was performed using a transnasal-transoral endoscopic approach. Postoperative radiotherapy was subsequently administered because of a positive surgical margin. As a result of this combined treatment, the patient has remained disease-free for three years following surgery. Although the final margin status was positive, the transnasal-transoral endoscopic approach provided an adequate surgical field, and adjuvant radiotherapy proved effective in achieving local disease control.

## Case presentation

**Figure 1:**
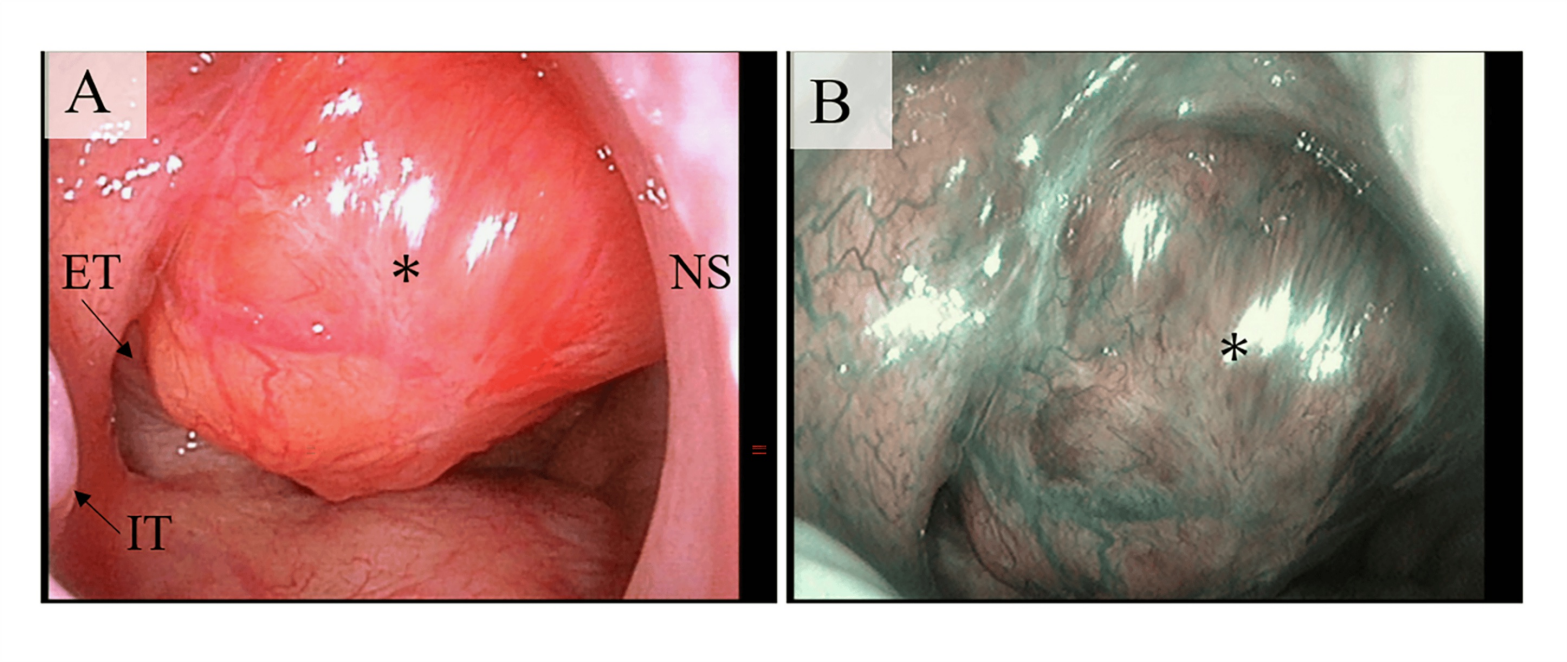
Endoscopic findings at the initial examination. (A) Endoscopic view via the right nasal cavity showed a submucosal tumor overlying the right Eustachian tube on the posterior wall of the nasopharynx (*). (B) Narrow-band imaging demonstrated no abnormal mucosal findings on the tumor surface, such as the absence of irregular microvascular patterns. ET: Eustachian tube; IT: inferior turbinate; NS: nasal septum.

An 80-year-old man presented to our clinic with a one-week history of right-sided ear fullness. He was undergoing treatment for membranous nephropathy with an estimated glomerular filtration rate (eGFR) of 16 mL/min, corresponding to stage 4 chronic kidney disease (eGFR 15-29 mL/min/1.73 m²) [3]. Otoscopic and nasopharyngeal examinations revealed right-sided serous otitis media and a submucosal mass on the posterior wall of the nasopharynx (Figure 1).

**Figure 2:**
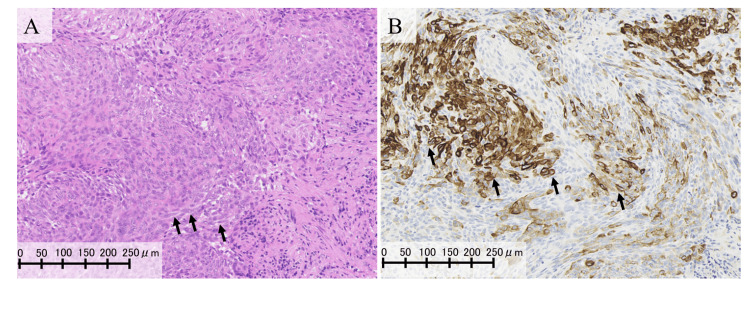
Histopathological and immunohistochemical findings of the tumor. (A) Microscopically, short spindle-shaped to spindle-shaped tumor cells were observed. Arrows indicate representative examples of spindle-shaped tumor cells. (B) Immunohistochemically, the tumor cells were partially positive for cytokeratin. Arrows indicate representative examples of cytokeratin-positive tumor cells.

Histopathological analysis of the biopsy specimen revealed squamous cell carcinoma with cytokeratin-positive spindle-shaped cells (Figure 2).

**Figure 3:**
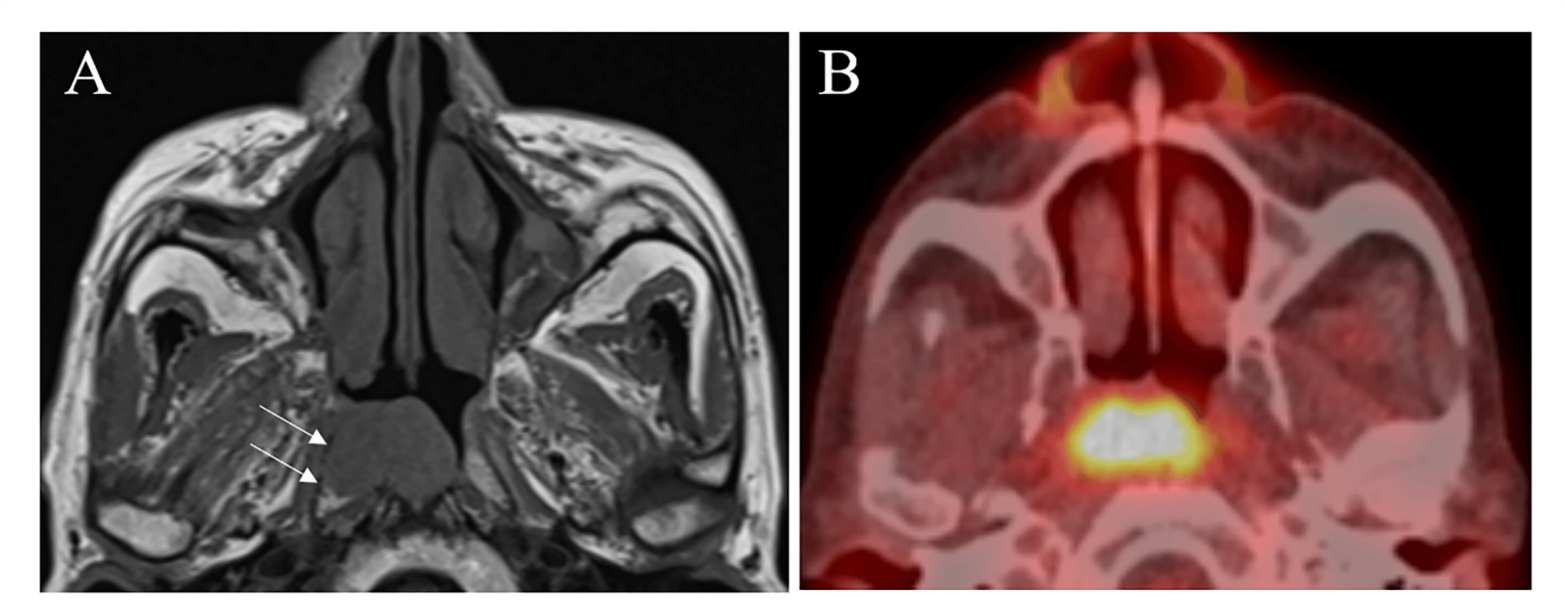
Imaging studies at the initial examination. (A) T1-weighted MRI showed the tumor was in contact with the right Eustachian tube (arrow). (B) PET-CT showed FDG uptake with a maximum standardized uptake value (SUVmax) of 9.8 in the tumor.

Magnetic resonance imaging (MRI) showed a 25-mm tumor on the posterior wall of the nasopharynx that was in contact with the right Eustachian tube cartilage (Figure 3A). A gadolinium contrast agent was not administered because of the patient’s renal dysfunction. Positron emission tomography-computed tomography (PET-CT) demonstrated fluorodeoxyglucose (FDG) uptake in the nasopharynx without evidence of regional or distant metastasis (Figure 3B).

**Figure 4:**
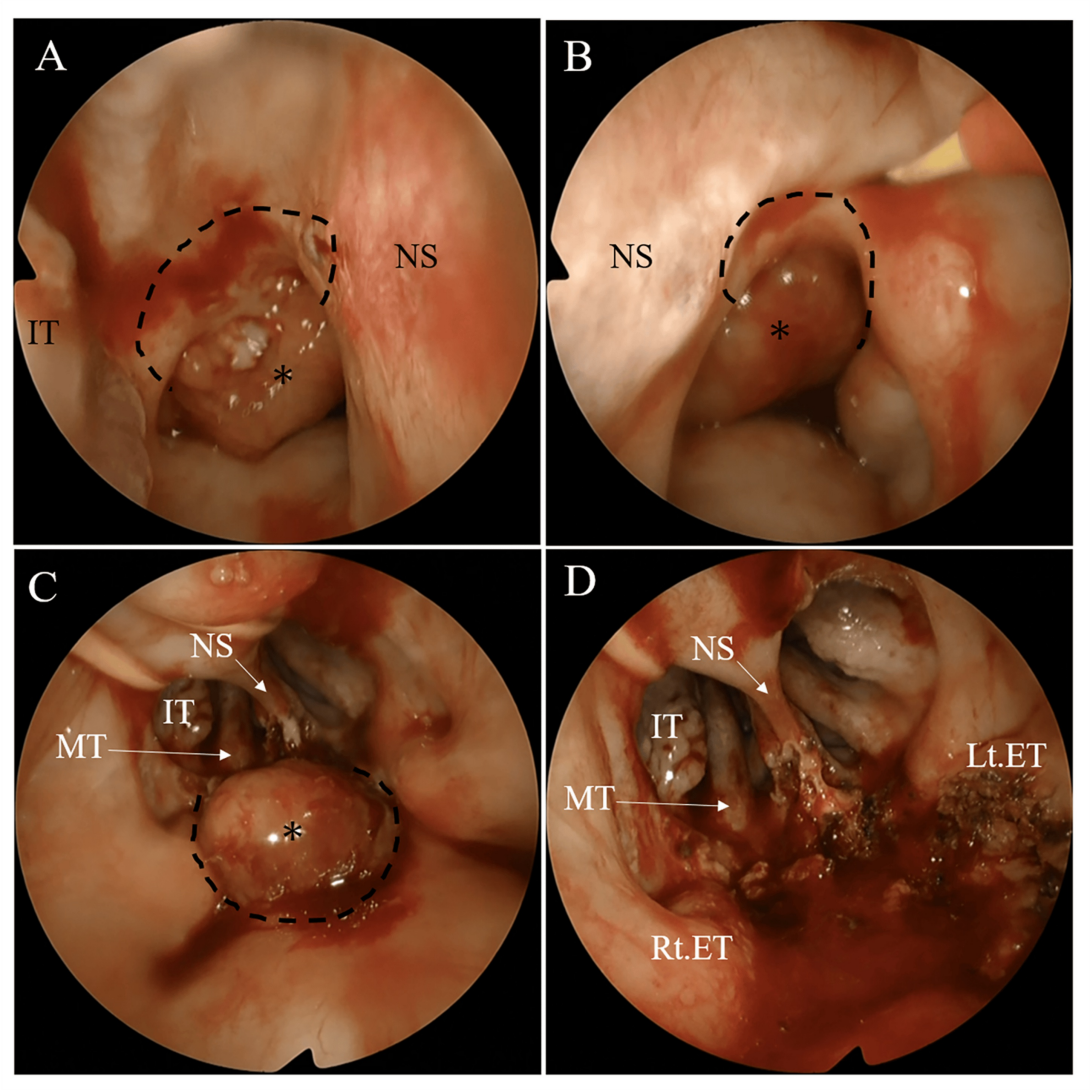
Intraoperative findings of transnasal–transoral approach. (A) Transnasal view via the right nasal cavity. The curved incision (dotted lines) was made around the tumor (*), from the posterior end of the nasal septum (NS) to the medial aspect of the inferior turbinate. (B) Transnasal view via the left nasal cavity. An incision (dotted lines) was made around the tumor (*) in the same manner on the right side. (C) Transoral view using a 70-degree endoscope. The curved incision (dotted lines) was made around the tumor (*) at the posterior wall of the nasopharynx. (D) Transoral view after resecting the tumor. On visual inspection, the Eustachian tube appeared intact. ET: Eustachian tube; IT: inferior turbinate; MT: middle turbinate; NS: nasal septum, Lt: left; Rt: right.

Based on these findings, the tumor was diagnosed as nasopharyngeal SpCC (T1N0M0). Given the limited efficacy of radiotherapy alone against the mesenchymal component of SpCC, surgical resection was planned using a transnasal endoscopic approach combined with a transoral approach as the tumor extended toward the oropharyngeal side. A transpterygoid approach is also considered if infiltration into the right Eustachian tube is identified during surgery [4,5]. However, because the patient’s severe chronic kidney disease limited his tolerance to prolonged surgery and large fluid shifts, maximal cytoreductive endoscopic resection was planned to reduce the tumor burden while minimizing surgical invasiveness, followed by postoperative radiotherapy. During surgery, a submucosal resection of the inferior turbinate was performed to make the surgical instruments easier to handle. The tumor was initially dissected anteriorly, cranially, and laterally from the distally located superior pharyngeal constrictor muscles (Figures 4A, 4B). Subsequently, posterior, caudal, and lateral dissections were performed using a 70-degree endoscope via the oropharynx (Figure 4C). This approach confirmed that the right Eustachian tube remained intact (Figure 4D), and intraoperative frozen section analysis demonstrated no malignant involvement of the Eustachian tube mucosa. The operative time was 121 min, the intraoperative infusion volume was 900 mL, the urine output was 300 mL, and the estimated blood loss was 150 mL.

**Figure 5:**
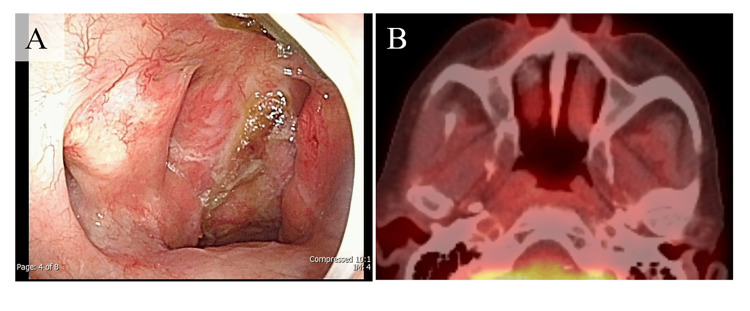
Endoscopic and radiologic findings three years after postoperative radiotherapy. (A) Endoscopic examination revealed no evidence of local recurrence. (B) PET–CT showed no evidence of recurrence.

Postoperatively, no intravenous fluids were administered, oral intake of water was resumed 3 hours after surgery, and the patient was discharged on postoperative day three. The final histopathological examination confirmed SpCC with a positive surgical margin on the posterior wall of the nasopharynx and a negative margin on the right side of the Eustachian tube. Thirty days after the surgery, localized intensity-modulated radiotherapy was administered to the nasopharynx, delivering a total dose of 65 Gy in 26 fractions. The clinical target volume included the postoperative tumor bed and area of the positive margin, with appropriate sparing of adjacent critical structures. The patient remained recurrence-free for >3 years after surgery (Figure 5).

Perioperative renal function remained stable (eGFR 15-18 mL/min). Although renal function gradually declined postoperatively, reaching an eGFR of 10 mL/min at 30 months and necessitating dialysis initiation, no acute deterioration in renal function was observed during radiotherapy.

## Discussion

In this case, postoperative radiotherapy administered for a positive surgical margin contributed to long-term disease-free survival, suggesting that adjuvant radiotherapy may play an important role in the management of SpCC with positive margins. The combined transnasal-transoral endoscopic approach provided an adequate surgical view, allowing maximal tumor resection with minimal invasiveness. Although the clinical benefit of surgery resulting in positive margins remains unclear, a strategy combining maximal cytoreductive resection and postoperative radiotherapy may be a viable treatment option for nasopharyngeal SpCCs in patients who cannot tolerate more invasive procedures. Further studies are required to establish optimal treatment strategies for patients with nasopharyngeal SpCC.

Advances in endoscopic techniques have improved surgical visualization and expanded the operative field, thereby facilitating minimally invasive tumor resection for nasopharyngeal malignancies. Two primary endoscopic approaches have been described for the treatment of localized early-stage nasopharyngeal carcinoma: transnasal and transoral approaches. The transnasal approach provides limited access to lesions extending inferiorly beyond the soft palate into the oropharynx, whereas the transoral approach provides access to the nasopharyngeal region below the soft palate, thereby overcoming this limitation [6]. In this case, the combined transnasal-transoral approach enabled sufficient visualization for tumor resection. Retrospective pathological evaluation revealed a negative surgical margin in the right Eustachian tube, indicating that complete resection may have been achieved. From this perspective, postoperative radiotherapy may not have been strictly necessary; however, it was administered to ensure local disease control.

This case highlights the potential therapeutic role of radiotherapy for SpCC. A previous cohort study suggested that combined surgery and radiotherapy provided better survival outcomes than surgery alone for patients with head and neck SpCC [1]. A previous case series reported long-term disease-free survival exceeding 3 years in patients with positive surgical margins who underwent postoperative radiotherapy or chemoradiotherapy for head and neck SpCC [7,8]. In these reports, none of the patients had regional or distant metastases, including cases of early-stage palatine tonsil and maxillary cancers as well as advanced ethmoid sinus cancer, suggesting that radiotherapy may be effective even in locally advanced SpCC. Conversely, the effectiveness of radiotherapy for SpCC has been inconsistent across observational studies. For early-stage laryngeal SpCC, favorable outcomes with low recurrence rates have been reported in patients treated with radiotherapy alone [9], whereas another study demonstrated better survival outcomes in patients treated with surgery alone than in those who underwent surgery followed by adjuvant radiotherapy [10]. However, the latter study acknowledged that radiotherapy was preferentially administered in more advanced cases, limiting definitive conclusions regarding its efficacy. Although SpCC has traditionally been considered relatively radioresistant, there is currently no conclusive evidence demonstrating the lack of benefit of radiotherapy for this disease.

In this case, maximal cytoreductive surgery was performed, followed by postoperative radiotherapy. The therapeutic rationale for cytoreductive surgery is supported by evidence from other solid tumors, in which tumor debulking enhances the effectiveness of adjuvant therapies. In advanced ovarian carcinoma, many patients present with extensive tumor dissemination throughout the abdomen and pelvis, making complete surgical resection challenging. In particular, when the disease involves the upper abdomen, achieving complete resection is generally difficult. Consequently, cytoreductive surgery followed by chemotherapy has become the standard treatment strategy and has been shown to confer significant survival benefits [11]. This benefit is attributed to a reduction in tumor burden, which decreases the number of treatment-resistant cells and limits the emergence of resistant phenotypes [12]. However, evidence supporting the effectiveness of cytoreductive surgery for SpCC remains limited, and it is possible that radiotherapy alone may have been sufficient in this case. Ideally, complete resection should be performed whenever feasible rather than cytoreductive surgery.

CKD is a major public health concern characterized by the progressive deterioration of renal function, with severity classified from stages 1 to 5 [3]. Patients with stage 4 CKD are at an increased risk of further renal impairment and cardiovascular complications due to surgical stress, anesthesia, and perioperative fluid and electrolyte fluctuations [3]. Although no definitive criteria exist regarding the acceptable operative duration or blood loss in such patients, minimizing surgical invasiveness and anesthesia exposure is essential. In the present case, perioperative management was successful, and the patient’s renal function remained stable during the perioperative period.

## Conclusions

This study demonstrates that a treatment strategy combining maximal cytoreductive endoscopic surgery with subsequent radiotherapy may be a feasible option for nasopharyngeal SpCC. This approach may be particularly beneficial for patients with significant comorbidities, such as advanced CKD, who are unable to tolerate extensive surgery. Larger studies and multicenter case series are needed to better define optimal management strategies, evaluate long-term outcomes, and clarify the respective roles of surgery and radiotherapy in nasopharyngeal SpCC. Future research should also focus on elucidating the biological behavior of nasopharyngeal SpCC and identifying prognostic factors to guide personalized treatment strategies.
